# MicroRNA-223 Increases the Sensitivity of Triple-Negative Breast Cancer Stem Cells to TRAIL-Induced Apoptosis by Targeting HAX-1

**DOI:** 10.1371/journal.pone.0162754

**Published:** 2016-09-12

**Authors:** Xu Sun, Yongqing Li, Meizhu Zheng, Wenshu Zuo, Wenzhu Zheng

**Affiliations:** 1 Department of Gastrointestinal Surgery, Shandong Cancer Hospital Affiliated to Shandong University, Shandong Academy of Medical Sciences, Jinan, 250117, China; 2 Breast Cancer Center, Shandong Cancer Hospital Affiliated to Shandong University, Shandong Academy of Medical Sciences, Shandong Academy of Medical Sciences, Jinan, 250117, China; 3 Emergency Medicine, Jinan Lixia District People's Hospital, Jinan, 250000, China; University of South Alabama, UNITED STATES

## Abstract

Drug resistance remains a significant challenge in the treatment of triple-negative breast cancer (TNBC). Recent studies have demonstrated that this drug resistance is associated with a group of cells known as cancer stem cells (CSCs), which are believed to determine the sensitivity of tumor cells to cancer treatment. MicroRNAs (miRNAs) are small, non-coding RNAs that play significant roles in normal and cancer cells. MiR-223 reportedly acts as a tumor suppressor in a range of cancers. However, the role of miR-223 in TNBC, especially in triple-negative breast cancer stem cells (TNBCSCs), remains unknown. Here, we found that miR-223 expression was down-regulated in CD44^+^CD24^-/low^ TNBCSCs compared with non-CSCs. Furthermore, we found that miR-223 overexpression resensitized TNBCSCs to tumor necrosis factor-related apoptosis-inducing ligand (TRAIL)-induced apoptosis. The HAX-1 gene, which is located in the mitochondria and functions as an anti-apoptotic protein, was found to be directly regulated by miR-223 in MDA-MB-231 cells. We demonstrated that miR-223 overexpression promoted TRAIL-induced apoptosis through the mitochondria/ROS pathway. In conclusion, our results suggest that miR-223 increases the sensitivity of TNBCSCs to TRAIL-induced apoptosis by targeting HAX-1. Our findings have improved our understanding of the role of miR-223 in TNBC and may contribute to TNBC treatment.

## Introduction

Breast cancer (BC) is the most common cancer in women worldwide and has a serious impact on women’s health [1]. Triple-negative breast cancer (TNBC) is a subtype of BC characterized by a high degree of malignancy and high incidences of recurrence and metastasis [2]. Because TNBC cells lack common therapeutic targets [3], chemotherapy and biotherapy are the only available treatments for TNBC [4]. Unfortunately, the repeated clinical medication of chemotherapeutic drugs usually induced the resistance of TNBC cells to these treatments, which leads to the tumor relapses [5]. Cancer stem cells (CSCs) are a group of cancer cells with the ability to self-renew and differentiate like normal stem cells [6]. Previous studies have demonstrated that CSCs are associated with treatment failure and tumor relapse [7]. Therefore, we isolated triple-negative breast cancer stem cells (TNBCSCs) and used the CD44^+^/CD24^-/low^ phenotype as a surface marker [8] to investigate the sensitivity of TNBCSCs to TNF-related apoptosis-inducing ligand (TRAIL).

TRAIL is part of the TNF superfamily, the members of which are expressed mainly by cells of the immune system [9]. TRAIL selectively triggers extrinsic and intrinsic apoptosis in tumor cells without influencing the function of normal cells [10]. Therefore, TRAIL is considered a promising anticancer agent with low toxicity and limited side effects. However, its therapeutic efficacy is severely compromised in cancer cells (especially CSCs) that exhibit low sensitivity to TRAIL-induced apoptosis [11]. Therefore, it is important to identify the mechanisms underlying this lack of sensitivity and to develop strategies that increase the sensitivity of CSCs to TRAIL.

MicroRNAs (miRNAs) are endogenous, small non-coding RNAs with less than 25 nucleotides. They can negatively regulate target gene expression by binding to the 3′-untranslated region (3′-UTR) of target mRNAs, which results in mRNA degradation or translational inhibition [12,13]. Previous studies have demonstrated that miRNAs regulate a wide array of biological processes, including cell proliferation, metastasis, differentiation, and apoptosis [14,15]. In addition, miRNA dysregulation has been linked the development of drug resistance in breast cancer [16,17]. However, the function of miR-223 in TNBCSCs remains unclear. In this study, we found that miR-223 expression was significantly decreased in triple-negative breast cancer stem cells. Furthermore, we demonstrated that increasing miR-223 expression improved the sensitivity of TNBCSCs to TRAIL.

## Results

### MiR-223 is down-regulated in triple-negative breast cancer stem cells

**Fig 1:**
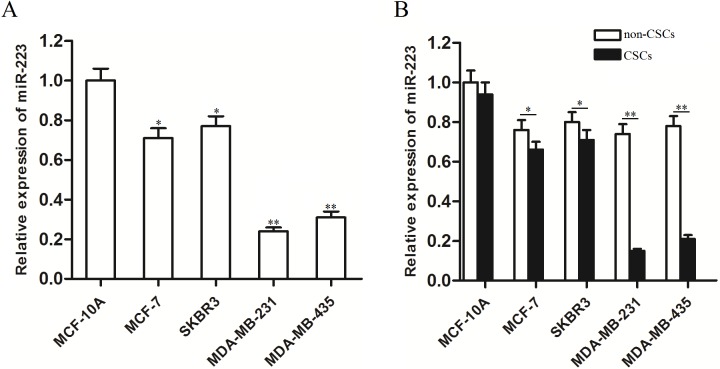
MiR-223 expression levels in breast cancer cell lines. (A) QRT-PCR analysis showed that the decrease of miR-223 expression was more significant in TNBC cell lines (MDA-MB-231 and MDA-MB-435) than the non-TNBC cell lines (MCF-7 and SKBR3). *P<0.05 *vs*. MCF-10A cells, t test, **P<0.01 *vs*. MCF-10A cells, t test. (B) MiR-223 expression was significantly down-regulated in both MDA-MB-231 CSCs and MDA-MB-435 CSCs compared with their parental cells. *P<0.05, t test, **P<0.01, t test.

To evaluate the basal expression levels of miR-223 in breast cancer cells and normal breast epithelial cells, we used malignant MCF-7, SKBR3, MDA-MB-231 and MDA-MB-435 cells and non-malignant MCF-10A cells. qRT-PCR results indicated that miR-223 expression was down-regulated in all of these breast cancer cell lines. However, the decrease of miR-223 expression was more significant in TNBC cell lines (MDA-MB-231 and MDA-MB-435, *P*<0.05 compared with the MCF-10A) than the non-TNBC cell lines (MCF-7 and SKBR3, *P*<0.01 compared with the MCF-10A) (Fig 1A). We next evaluated the basal expression levels of miR-223 in stem cells obtained from these mentioned cells. For cell sorting of stem cells, MCF-10A, MCF-7,SKBR3,MDA-MB-231 and MDA-MB-435 cell lines were incubated with anti–CD24-FITC and anti–CD44-PE antibodies on ice for 40 min in the dark. After being washed with cold PBS, CD44^+^CD24^−/low^ cells were purified by flow cytometry as the stem cells, and the rest cells were considered as the non-stem cells. The results of qRT-PCR analysis showed significant decrease of miR-223 expression in both MDA-MB-231 CSCs and MDA-MB-435 CSCs compared with their parental cells (*P*<0.01). Meanwhile the differences between MCF-10A, MCF-7,SKBR3-stem cells and non-stem cells were slighter (*P*<0.05) (Fig 1B). These results indicate that miR-223 is down-regulated in TNBCSCs.

### TNBCSCs are resistant to TRAIL compared with non-cancer stem cells (non-CSCs)

**Fig 2:**
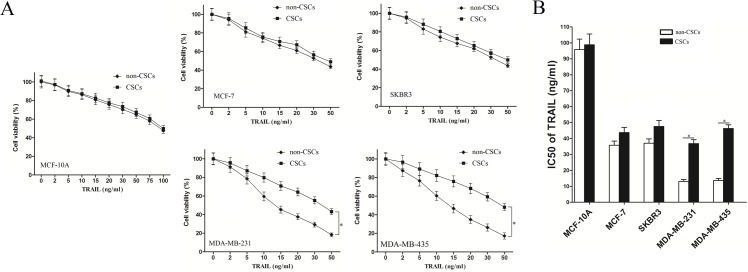
TNBCSC and non-CSC sensitivity to TRAIL treatment. (A) Sensitivity of MCF-10A and breast cancer stem cells and non-stem cells to TRAIL was determined via MTT assays. *P<0.05, t test. (B) The IC50 of TRAIL was determined according to the cell viability curves calculated based on the MTT assay results. *P<0.05, t test.

To investigate the sensitivity of breast cancer stem cells and non-breast cancer stem cells to TRAIL, we sorted CSCs and non-CSCs from the MCF-10A, MCF-7, SKBR3, MDA-MB-231 and MDA-MB-435 cell lines and cultured them in DMEM. Subsequently, the MTT assays were performed. As the nonmalignant cells were not sensitive to TRAIL [10], we observed that MCF-10A could survive in higher concentration of TRAIL compared with the breast cancer cells (Fig 1A). Furthermore, the half-maximal inhibitory concentration (IC50) values of TRAIL were significantly higher in MDA-MB-231 CSCs and MDA-MB-435 CSCs than in non-CSCs (*P*<0.01). However, the differences of IC50 values of TRAIL were slight between the CSCs and non-CSCs in MCF-7 and SKBR3 cell lines (Fig 2B). These results are demonstrative of the resistance of TNBCSCs to TRAIL.

### TNBCSCs are sensitive to miR-223-mediated cell death induced by TRAIL

**Fig 3:**
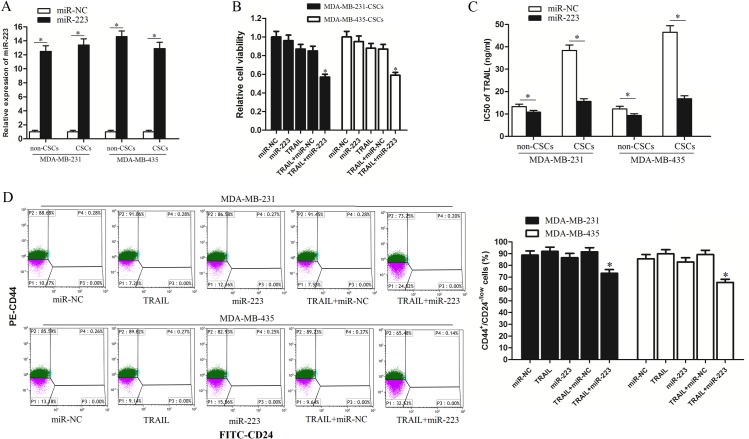
TNBCSCs are sensitive to miR-223-mediated cell death induced by TRAIL. (A) The transfection efficiency of miR-223 mimics was evaluated by qRT-PCR. *P<0.05, t test. (B) TNBCSCs were transfected with miR-223 mimics. Twenty-four hours after transfection, the cells were treated with TRAIL (10 ng/ml) for 48 h. Relative cell viability was determined by MTT assay. *P<0.05 *vs*. the TRAIL+miR-NC group, t test. (C) TNBCSCs or non-CSCs were transfected with miR-223 mimics. Twenty-four hours after transfection, the cells were treated with different concentrations of TRAIL for 48 h, and then MTT assays were performed. The IC50 of TRAIL was determined according to the cell viability curves calculated based on the MTT assay results. *P<0.05, t test. (D) The CD44^+^/CD24^-/low^ TNBCSC population was assessed by FACS. *P<0.05 *vs*. the TRAIL+miR-NC group, t test.

To determine the biological role of miR-223 in TRAIL treatment, tumor cells were transfected with miR-223 mimics. Because miR-223 was significantly overexpressed due to these transfections (Fig 3A), combination treatment with miR-223 mimics and TRAIL was administered. In addition, as the 10 ng/ml TRAIL induced slight cell death in both MDA-MB-231-CSCs and MDA-MB-435-CSCs (Fig 2A), we chose this concentration of TRAIL for combination treatment with miR-223 mimics. As shown in Fig 3B, although miR-223 treatment did not cause significant cytotoxicity, it significantly sensitized TNBCSCs to TRAIL-induced cell death. Furthermore, miR-223 transfection significantly decreased the IC50 of TRAIL in CSCs (59.4% in MDA-MB-231 and 63.9% in MDA-MB-435) compared with non-CSCs (18.8% in MDA-MB-231 and 22.9% in MDA-MB-435) (Fig 3C). We next evaluated the effects of miR-223 and TRAIL on CSCs by analyzing CD44 and CD24 expression via FACS. Interestingly, we found that TRAIL slightly increased the population of CD44^+^/CD24^-/low^ TNBCSCs, whereas the combination of TRAIL and miR-223 significantly decreased the population of CD44^+^/CD24^-/low^ TNBCSCs (Fig 3D). These results indicate that TNBCSCs are more sensitive to miR-223-mediated cell death induced by TRAIL than non-CSCs.

### HAX-1 is the target of miR-223 in TNBCSCs

**Fig 4:**
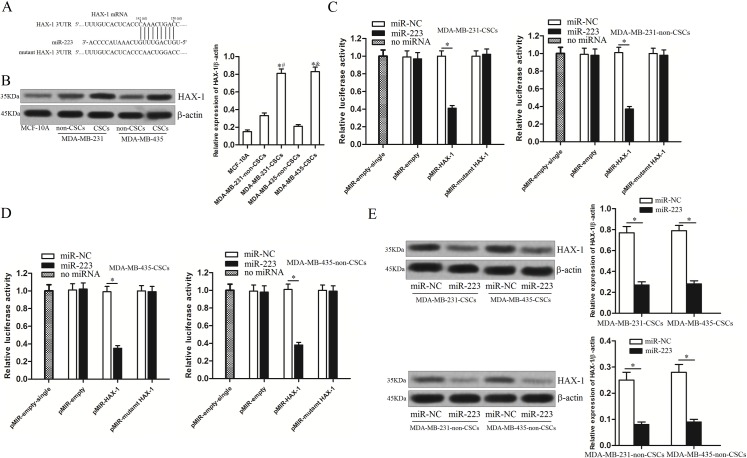
HAX-1 is the target of miR-223 in TNBCSCs. (A) HAX-1 was predicted to be a target of miR-223 by the TargetScan database. (B) HAX-1 expression in MDA-MB-231 CSCs, MDA-MB-231 non-CSCs, MDA-MB-435 CSCs, MDA-MB-435 non-CSCs, and MCF-10A cells was evaluated by western blot analysis. *P<0.05 *vs*. the MCF-10A, t test, ^#^*P*<0.05 *vs*. the MDA-MB-231-non-CSCs, t test, ^&^*P*<0.05 *vs*. the MDA-MB-435-non-CSCs, t test. (C) MDA-MB-231 CSCs or MDA-MB-231-non-CSCs were cotransfected with the wildtype/mutant 3’-UTR of HAX-1 and miR-223 mimics as indicated. Forty-eight hours post-transfection, luciferase activity was detected using a dual-luciferase reporter assay system, according to the manufacturer’s instructions. *P<0.05, t test. (D) Luciferase assays in MDA-MB-435 CSCs or MDA-MB-231-non-CSCs. *P<0.05, t test. (E) Western blot analysis showed that transfection of miR-223 mimics down-regulated HAX-1 expression in TNBCSCs. *P<0.05, t test.

To determine how miR-223 facilitates TRAIL-induced apoptosis in TNBCSCs, the TargetScan database (http://www.targetscan.org/) was used to predict the targets of miR-223. Of the target genes predicted by this database, the HAX-1 (hematopoietic cell-specific protein 1-associated protein X-1) gene was considered one of the most likely targets (Fig 4A) because it is an important regulator of the mitochondrial apoptosis pathway [18]. We therefore evaluated HAX-1 expression in TNBC cells. As shown in Fig 4B, we observed that HAX-1 expression levels were significantly higher in CSCs than in non-CSCs and MCF-10A cells (Fig 4B). As miR-223 is down-regulated in TNBCSCs (Fig 1B), there was a negative correlation between miR-223 and HAX-1. To investigate the hypothesis that HAX-1 is the target of miR-223 in TNBC, we cloned HAX-1 3’-UTR sequences containing predicted miR-223 target sites into a luciferase reporter vector. Luciferase assays revealed that the miR-223 mimics significantly decreased luciferase activity in the wild-type HAX-1 3’-UTR, whereas mutation of the putative miR-223 target sites in the HAX 1 3’-UTR abrogated the responsiveness of luciferase to miR-223 (Fig 4C and 4D). To confirm that miR-223 regulates HAX-1 expression, western blot analysis was performed to measure HAX-1 protein levels after TNBC cells were transfected with miR-223 mimics. As shown in Fig 4E, miR-223 mimics significantly decreased HAX-1 expression in MDA-MB-231 CSCs (or non-CSCs) and MDA-MB-435 CSCs (or non-CSCs). Taken together, these results indicate that the HAX-1 gene is a functional target of miR-223 in TNBC.

### MiR-223-mediated cell death induced by TRAIL is dependent on HAX-1 down-regulation

**Fig 5:**
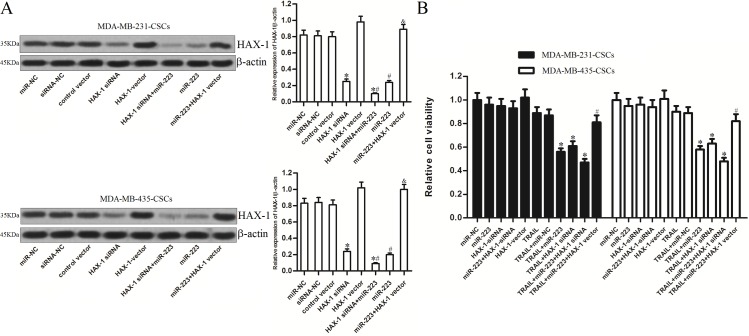
MiR-223 promoted TRAIL-induced cell death by down-regulating HAX-1 expression in TNBCSCs. (A) The transfection efficiency of HAX-1 siRNA and HAX-1 vector in MDA-MB-231 CSCs and MDA-MB-435 CSCs was evaluated by western blot analysis. *P<0.05 *vs*. the siRNA-NC group, t test, ^#^*P*<0.05 *vs*. the miR-NC group, t test, ^&^*P*<0.05 *vs*. the miR-223 group, t test. (B) MDA-MB-231 CSCs and MDA-MB-435 CSCs were transfected with miR-223 mimics, HAX-1 siRNA, and HAX-1 vector. Twenty-four hours after transfection, the cells were treated with TRAIL (10 ng/ml) for 48 h. Relative cell viability was determined by MTT assay. *P<0.05 *vs*. the TRAIL+miR-NC group, ^#^*P*<0.05 *vs*. the TRAIL+miR-223 group, t test.

As HAX-1 was proven to be the target of miR-223 in TNBCSCs, we changed the expression levels of HAX-1 using a specific siRNA and a recombinant eukaryotic expression vector (transfection efficiency is shown in Fig 5A). We observed that both miR-223 mimics and HAX-1 siRNA significantly promoted TRAIL-induced cell death in TNBCSCs. In contrast, enforced expression of HAX-1 via the abovementioned recombinant plasmid abolished the synergistic effects of TRAIL and miR-223 (Fig 5B). These results indicate that miR-223 overexpression facilitates TRAIL-induced cell death by down-regulating HAX-1 expression.

### MiR-223 increases TRAIL sensitivity via a caspase-dependent apoptotic pathway

**Fig 6:**
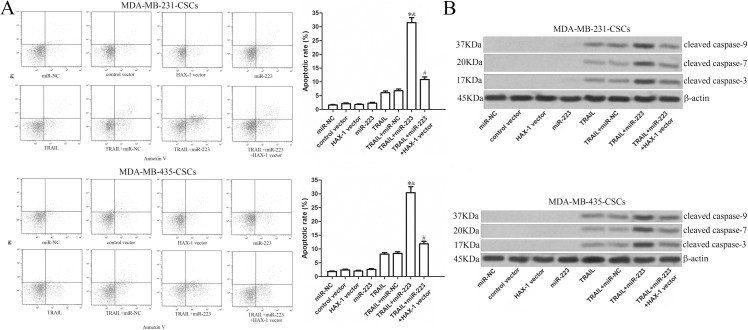
MiR-223 increased TRAIL sensitivity via a caspase-dependent apoptotic pathway. (A) MDA-MB-231 CSCs and MDA-MB-435 CSCs were transfected with miR-223 mimics and HAX-1 vector. Twenty-four hours after transfection, the cells were treated with TRAIL (10 ng/ml) for 48 h. Flow cytometry analysis was performed to measure the cell apoptosis. *P<0.05 *vs*. the TRAIL+miR-NC group, ^&^*P*<0.05 *vs*. the TRAIL group, ^#^*P*<0.05 *vs*. the TRAIL+miR-223 group, t test. (B) MDA-MB-231 CSCs and MDA-MB-435 CSCs were transfected with miR-223 mimics and HAX-1 vector. Twenty-four hours after transfection, the cells were treated with TRAIL (10 ng/ml) for 48 h. Western blot analysis was performed to detect caspase-9, caspase-7 and caspase-3 activation cleavage.

To study the pathway by which miR-223 enhances TRAIL-induced cell death in TNBCSCs, treated cells were harvested and stained with Annexin V and PI. As shown in Fig 6A, the apoptosis induced by the combination of miR-223 and TRAIL was significantly stronger than that induced by miR-223 or TRAIL alone. In addition, HAX-1 siRNA transfection inhibited the apoptosis induced by the combination of miR-223 and TRAIL. Meanwhile, as shown in Fig 6B, miR-223 plus TRAIL triggered caspase-9, caspase-7, caspase-3 activation. These findings demonstrate that caspase-9, caspase-7, and caspase-3 activation is associated with miR-223-mediated TRAIL-induced apoptosis.

### Combined treatment with miR-223 and TRAIL induces ROS production and mitochondrial dysfunction in TNBCSCs

**Fig 7:**
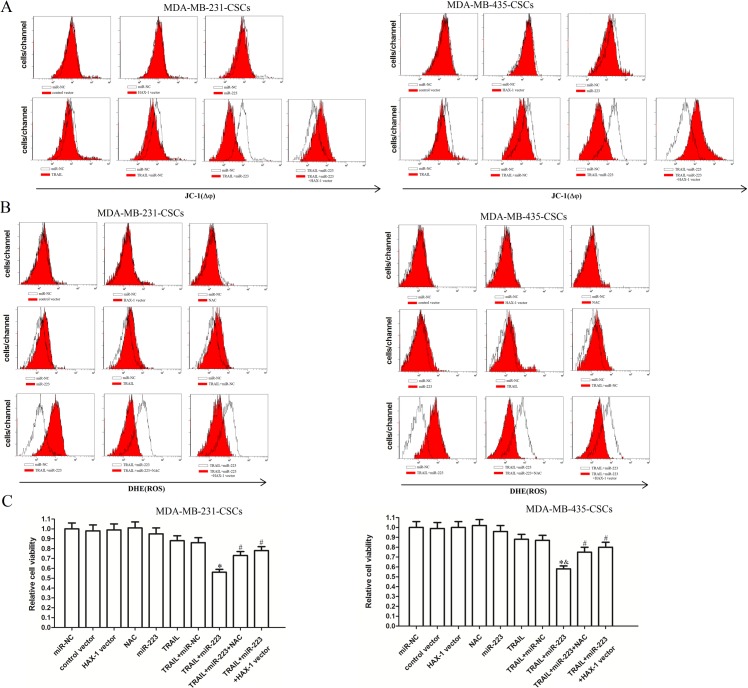
MiR-223 promoted TRAIL-induced apoptosis through the mitochondria/ROS pathway. (A) The mitochondrial membrane potential (ΔΨ_m_) of MDA-MB-231 CSCs and MDA-MB-435 CSCs cells treated with miR-223 and TRAIL was detected using JC-1 staining and flow cytometry. (B) ROS generation was detected using DHE staining and flow cytometry. (C) MDA-MB-231 CSCs and MDA-MB-435 CSCs cells were treated with miR-223 and TRAIL (10 ng/ml) in the presence or absence of 5 mM NAC. Cell viability was then detected by MTT assay. *P<0.05 *vs*. the TRAIL+miR-NC group, ^&^*P*<0.05 *vs*. the TRAIL group, ^#^*P*<0.05 *vs*. the TRAIL+miR-223 group, t test.

HAX-1 is the target of miR-223 and is an important inhibitor of the mitochondrial apoptosis pathway [18]; therefore, we measured the mitochondrial membrane potential of TNBCSCs via JC-1 staining. As expected, although miR-223 alone did not significantly influence ΔΨ_m_, it significantly facilitated TRAIL-induced mitochondrial damage in MDA-MB-231 CSCs and MDA-MB-435 CSCs (Fig 7A). In addition, HAX-1 vector transfection protected the mitochondria from damage induced by co-treatment with miR-223 and TRAIL, demonstrating the important role of HAX-1 in mitochondrial apoptosis. Previous studies have shown that ROS generation can be induced by mitochondrial dysfunction [19]. Therefore, DHE staining was used to assess the effects of miR-223 and TRAIL on ROS generation. Consistent with previous results, the combination of miR-223 and TRAIL induced significant ROS generation, which was inhibited by either the HAX-1 vector or N-acetylcysteine (NAC), a strong scavenger of ROS [20] (Fig 7B). The involvement of ROS in miR-223-mediated cell death was subsequently investigated. As shown in Fig 7C, both the HAX-1 vector and NAC abolished the synergistic effects of TRAIL and miR-223 on TNBCSCs. Taken together, these results indicate that miR-223 overexpression promotes TRAIL-induced apoptosis through the mitochondria/ROS pathway.

### MiR-223 enhances the anti-tumor effect of doxorubicin and cisplatin

**Fig 8:**
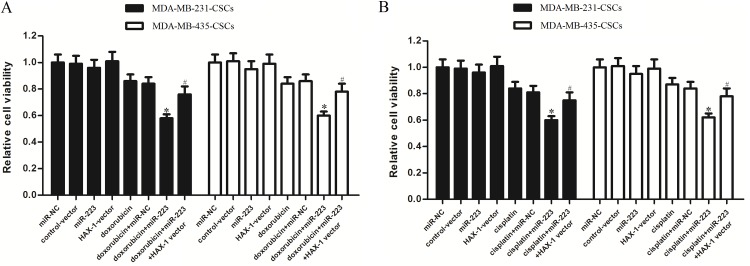
MiR-223 enhances the anti-tumor effect of doxorubicin and cisplatin in TNBCSCs. (A) MDA-MB-231 CSCs and MDA-MB-435 CSCs were transfected with miR-223 mimics and HAX-1 vector. Twenty-four hours after transfection, the cells were treated with doxorubicin (2 μg/ml) for 48 h. Relative cell viability was determined by MTT assay. *P<0.05 *vs*. the doxorubicin+miR-NC group, t test, ^#^*P*<0.05 *vs*. the doxorubicin+miR-223 group, t test. (B) MDA-MB-231 CSCs and MDA-MB-435 CSCs were transfected with miR-223 mimics and HAX-1 vector. Twenty-four hours after transfection, the cells were treated with cisplatin (2 μM) for 48 h. Relative cell viability was determined by MTT assay. *P<0.05 *vs*. the cisplatin+miR-NC group, t test, ^#^*P*<0.05 *vs*. the cisplatin+miR-223 group, t test.

As the preceding results indicated that the miR-223/HAX-1 axis regulates the TRAIL-induced apoptosis in TNBCSCs, we next investigated the responses of TNBCSCs to some other anti-tumor chemotherapeutic agents such as doxorubicin and cisplatin when they were transfected with miR-223. First of all, we chose 2 μg/ml doxorubicin and 2 μM cisplatin for treatment to TNBCSCs, because both of these concentrations of doxorubicin and cisplatin induced slight cell death of TNBCSCs when they were singly used. As shown in Fig 8A and 8B, we found that the introduction of miR-223 significantly increased the cytotoxicity of doxorubicin or cisplatin to MDA-MB-231-CSCs and MDA-MB-435-CSCs. Furthermore, the synergistic effect between miR-223 and doxorubicin or cisplatin was abolished by the enforced expression of HAX-1. These results indicated that miR-223/HAX-1 axis regulates the sensitivity of TNBCSCs to cytotoxic chemotherapeutic agents.

## Discussion

Recent studies have demonstrated that miRNAs are associated with cancer therapy efficiency [21,22]. In addition, miRNA dysregulation in CSCs is one of many factors responsible for cancer treatment failure [23,24]. MiR-223 reportedly acts as a tumor suppressor in multiple cancers. For example, miR-223 was shown to be down-regulated in cervical cancer. Enforced miR-223 expression inhibited cervical cancer cell metastasis by modulating epithelial-mesenchymal transition (EMT) [25]. In prostate cancer, miR-223 suppressed tumor cell proliferation, migration, and invasion by targeting ITGA3/ITGB1 (integrin subunit alpha 3/ integrin subunit beta 1) signaling [26]. In the present study, we demonstrated that miR-223 was significantly down-regulated in MDA-MB-231 CSCs and MDA-MB-435 CSCs, both of which are TNBCSCs. In addition, the combination of miR-223 and TRAIL was unexpectedly efficient with respect to facilitating TNBC cell apoptosis, especially TNBC CSC apoptosis. Furthermore, our results (Fig 2C and 2D) indicated that TNBCSCs are more sensitive to miR-223-mediated cell death induced by TRAIL than non-CSCs. These data indicate that miR-223, as well as its target genes, plays a role in mitigating TNBCSC resistance to TRAIL.

HAX-1 (hematopoietic cell-specific protein 1-associated protein X-1) is a 35 kDa protein, which locates in mitochondria, endoplasmic reticulum and cytoplasm. Pervious reports have demonstrated that HAX-1 could inhibit the activation of caspase-9 directly; besides, it could interact with the mitochondrial proteases Parl (presenilin-associated, rhomboid-like) and HtrA2 (high-temperature-regulated A2), and thereby preventing the accumulation of mitochondrial-outer-membrane-associated activated Bax (BCL2 associated X, apoptosis regulator), an event that initiates apoptosis. Therefore, it functions as an important anti-apoptotic protein through the mitochondrial pathway [27–30]. Studies have shown that HAX-1 protects cells from mitochondrial damage induced by drugs and environmental factors and decreases pro-apoptotic factor release from the mitochondria by attenuating the loss of ΔΨ_m_ [31,32]. Therefore, HAX-1 is usually overexpressed in many malignancies, including breast cancer [33]. Anti-cancer agents targeting HAX-1 may represent a new treatment strategy for TNBC [34]. In this paper, we found that the HAX-1 protein was overexpressed in TNBCSCs, which rendered it sensitive to miR-223 or HAX-1 siRNA. We subsequently demonstrated that miR-223 overexpression promoted TRAIL-dependent apoptosis by regulating HAX-1 expression directly. Consistent with these findings, knockdown of HAX-1 by its specific siRNA exerted effects on TRAIL-induced cell death similar to those of miR-223. All of these data are indicative of the role of the miR-223/HAX-1 interaction in TNBCSCs.

Apoptosis is an important target of cancer treatment and is usually induced by mitochondrial dysfunction [35]. Mitochondria damage results in the opening of a non-specific pore in the inner mitochondrial membrane known as the mPTP (mitochondrial permeability transition pore). Opening of the mPTP causes release of mitochondrial components into the cytoplasm [36]. Among these components are ROS, which are key inducers of mitochondrial apoptosis [37]. In our study, we demonstrated that miR-223-mediated apoptosis induced by TRAIL is dependent on mitochondrial collapse and ROS generation, followed by activation of effector caspases (such as caspase-9, caspase-7, and caspase-3).

In summary, we have provided strong evidence that miR-223 mediates TRAIL sensitivity in triple-negative breast cancer stem cells by regulating the anti-apoptotic HAX-1 gene. MiR-223/HAX-1-targeted therapy may represent a novel strategy for sensitizing TNBCSCs to clinical treatment.

## Materials and Methods

### Cell culture

MCF-10A cells, which are normal human breast epithelial cells [38], MCF-7, SKBR3, and the TNBC cell lines MDA-MB-231 and MDA-MB-435 were purchased from ATCC. All these cell lines were cultured according to the instruction provided by ATCC. Breast stem cells were isolated by sorting CD44^+^CD24^−/low^ populations using anti–CD24-FITC and anti–CD44-PE antibodies (BD Biosciences, USA). Briefly, MCF-10A, MCF-7,SKBR3,MDA-MB-231 and MDA-MB-435 cells were incubated with anti–CD24-FITC and anti–CD44-PE antibodies on ice for 40 min in the dark. After being washed with cold PBS, CD44^+^CD24^−/low^ cells were purified by flow cytometry (BD Biosciences).

### Quantitative reverse transcriptase real-time PCR (qRT-PCR)

RNA was extracted from MCF-10A, MCF-7, SKBR3, MDA-MB-231 and MDA-MB-435 cells using the TRIzol reagent (Invitrogen, USA), according to the manufacturer’s instructions. MiR-223 reverse transcription was performed using a stem-loop RT primer and the PrimeScript RT reagent Kit (Takara, China), The miR-223 RT primer (RiboBio Co. Ltd, Guangzhou, China) had the following sequence: 5'-CTCAACTGGTGTCGTGGAGTCGGCAATTCAGTTG AGTGGGGTAT-3'. qRT-PCR was performed in triplicate using SYBR Premix Ex Taq (TaKaRa), according to the manufacturer’s instructions. The miR-223 amplified primers had the following sequences: forward prime: 5'-ACACTCCAGCTGGGTGTCAGTTTGTCAAAT, reverse primer: TGGTGTCGTGGAGTCG. PCR was performed under the following thermal cycling conditions: 95°C for 30 sec, followed by 40 cycles of 95°C for 5 sec and 60°C for 30 sec, and one cycle of 95°C for 15 sec, 60°C for 60 sec and 95°C for 15 sec for dissociation. Analyses were based on the comparative Ct method (2^-ΔΔCT^) [39], and U6 snRNA served as the internal control.

### Transfection

MiR-223 mimics, negative controls (miR-NCs), control siRNA (siRNA-NC) and HAX-1 siRNA were synthesized by RiboBio Co. Ltd. (China). The HAX-1 eukaryotic expression vector was generated by cloning the open reading frame of the HAX-1 gene into a pcDNA3.1 vector (Life Technologies, USA). For miR-223 overexpression, 50 pmol/ml miR-223 mimics were transfected using Lipofectamine 2000 (Invitrogen), according to the manufacturer’s instructions. For gain- and loss-of-function studies involving the HAX-1 gene, 2 μg/ml HAX-1 vector and 50 pmol/ml HAX-1 siRNA were transfected using Lipofectamine 2000.

### Luciferase reporter assay

For the luciferase assay, the putative binding sites of miR-223 in the 3' UTR of the human HAX-1 gene were amplified and inserted into the luciferase reporter pMIR-Report-Vector (Life Technologies) downstream of the luciferase reporter gene. Mutations were introduced into the 3' UTR of HAX-1 mRNA (CAAACUGAC to CAACUGGAC) using a QuikChange Site-Directed Mutagenesis Kit (Stratagene, USA). The reporter vector plasmid and miR-223 mimics were co-transfected into TNBC cells using Lipofectamine 2000. Following transfection for 48 h, the cells were collected and lysed. Luciferase activity was then measured using the Dual Luciferase Reporter Assay System (Promega, USA), and luminescence was recorded on a Synergy Multi-Mode Plate Reader (Biotek, USA). Firefly luciferase activity was normalized to Renilla luciferase activity.

### Cell viability assay

For the cell viability assay, 4×10^3^ cells per well were seeded in 96-well plates and transfected with RNAs and plasmids. Twenty-four hours after transfection, the cells were treated with TRAIL, doxorubicin, or cisplatin for 48 h, and cell viability was evaluated via 3-(4, 5-dimethylthiazol-2-yl)-2, 5-diphenyltetrazolium bromide (MTT) assay. The absorbance was read at 570 nm using a microplate reader (Sunrise Microplate Reader; TECAN).

### Western blot analysis

Total protein was extracted using RIPA buffer. Whole-cell lysates were separated via 12.5% sodium dodecyl sulfate polyacrylamide gel electrophoresis (SDS-PAGE) and transferred to a PVDF membrane (Millipore, USA). Non-specific binding was blocked by incubating the membranes in 5% skim milk for 1 h at room temperature. The membranes were incubated overnight at 4°C with the appropriate primary antibodies (cleaved caspase-9, cleaved caspase-7, cleaved caspase-3, and β-actin were purchased from Cell Signaling Technology, USA;HAX-1 was purchased from Santa Cruze Biotechnology, USA). Blots were then detected with an enhanced chemiluminescence detection kit (Pierce, USA) and shown in S1 File.

### Apoptosis assay

Approximately 5×10^5^ cells per well were seeded in 6-well plates and transfected with RNAs and plasmids. Twenty-four hours after transfection, the cells were treated with 10 ng/ml TRAIL for 48 h, and cell apoptosis was measured via FITC-Annexin V and PI staining. The percentage of apoptotic cells was quantified using flow cytometry.

### Measurement of reactive oxygen species (ROS)

Approximately 5×10^5^ cells per well were seeded in 6-well plates and transfected with RNAs and plasmids. Twenty-four hours after transfection, the cells were treated with 10 ng/ml TRAIL for 48 h, and ROS generation was measured via dihydroethidium (DHE, Molecular Probes, USA) staining and flow cytometry, according to the manufacturer’s instructions.

### Measurement of mitochondrial membrane potential (MMP, ΔΨ_m_)

For ΔΨ_m_ measurement, 5×10^5^ cells per well were seeded in 6-well plates and transfected with RNAs and plasmids. Twenty-four hours after transfection, the cells were treated with 10 ng/ml TRAIL for 48 h, and ΔΨ_m_ was measured using 5,5',6,6'-Tetrachloro-1,1',3,3'-tetraethyl-benzimidazolyl-carbocyanine iodide (JC-1, Molecular Probes) staining and flow cytometry, according to the manufacturer’s instructions.

### Statistical analysis

All data are presented as the mean ± standard deviation of three independent experiments. The statistical significance of the differences between groups was determined using Student’s t test, and all analyses were performed using SPSS 14.0 software. *P*<0.05 was considered statistically significant.

## Supporting Information

S1 File The blots of mentioned proteins. (ZIP)
