# Supplementary figures and images for: MicroRNA-223 Increases the Sensitivity of Triple-Negative Breast Cancer Stem Cells to TRAIL-Induced Apoptosis by Targeting HAX-1
Source: PLoS One. 2016 Sep 12;11(9):e0162754. doi: 10.1371/journal.pone.0162754 (PMC5019415; doi:10.1371/journal.pone.0162754)

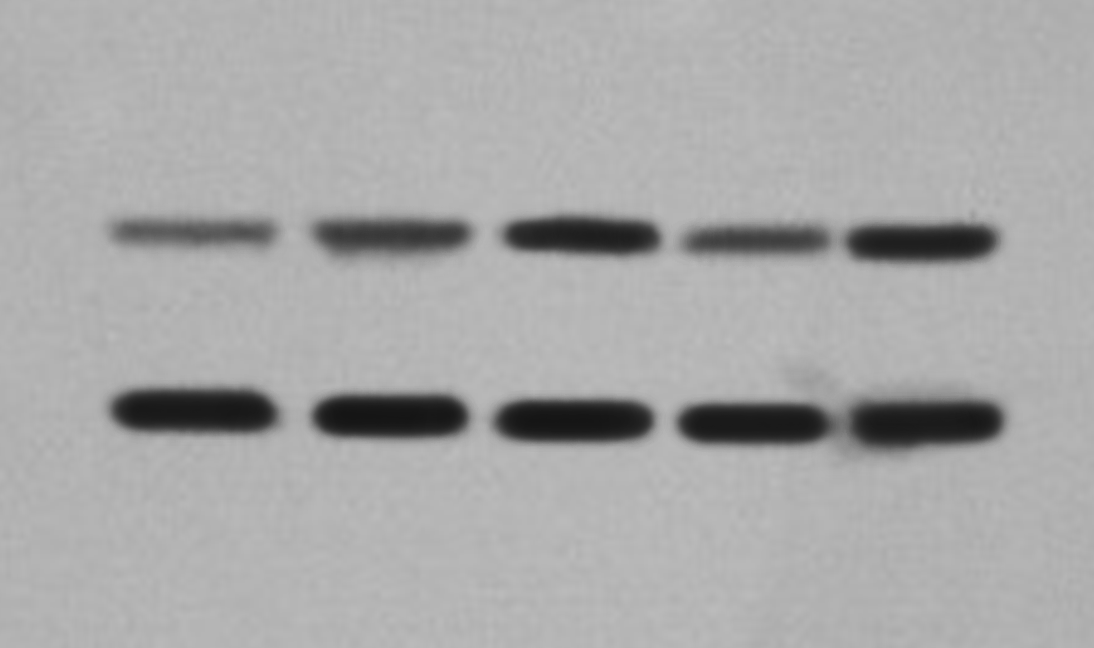

Supplement: S1 File — (ZIP) [file pone.0162754.s001.zip › western blotting/Fig4B.tif]

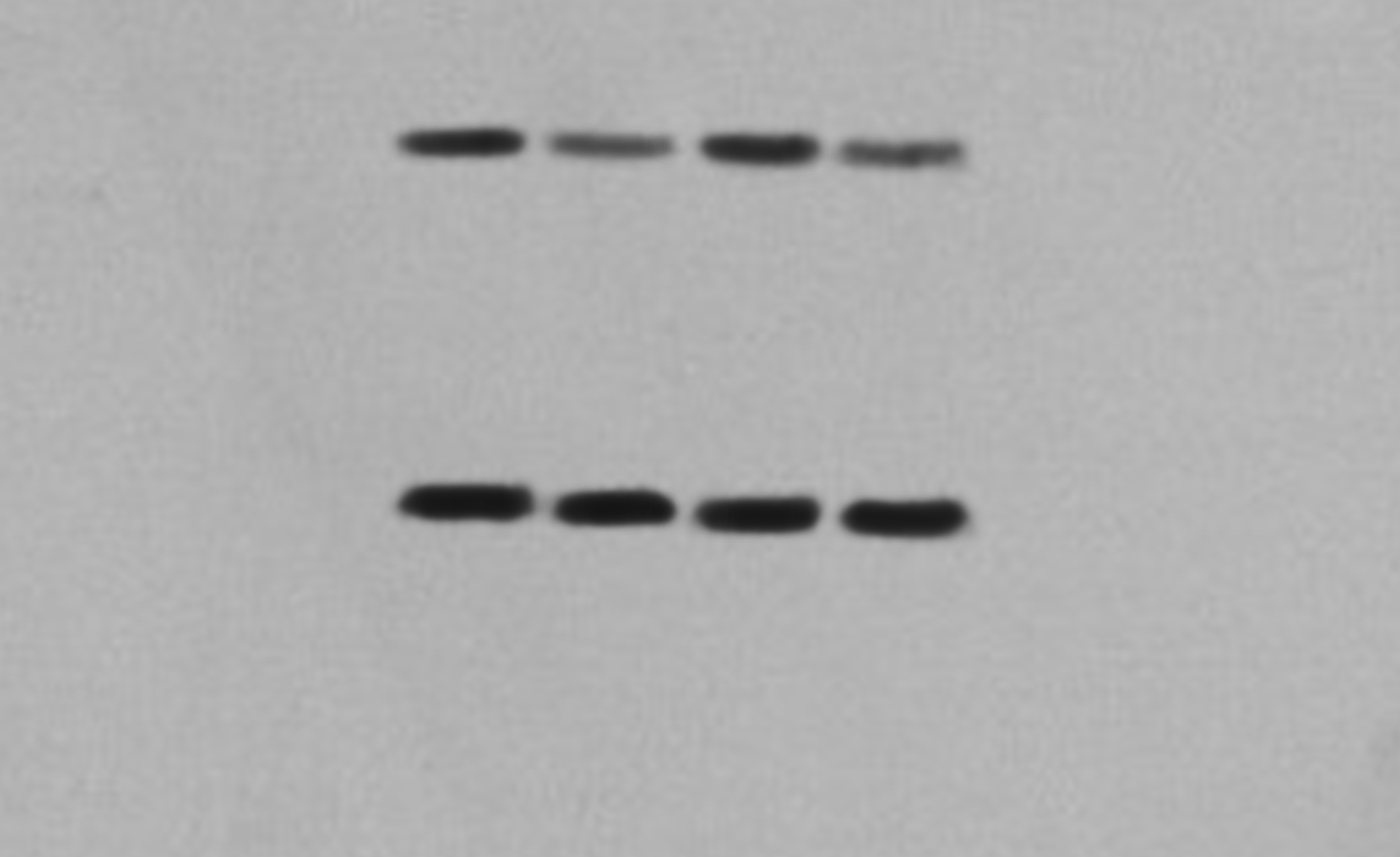

Supplement: S1 File — (ZIP) [file pone.0162754.s001.zip › western blotting/Fig4E-1.tif]

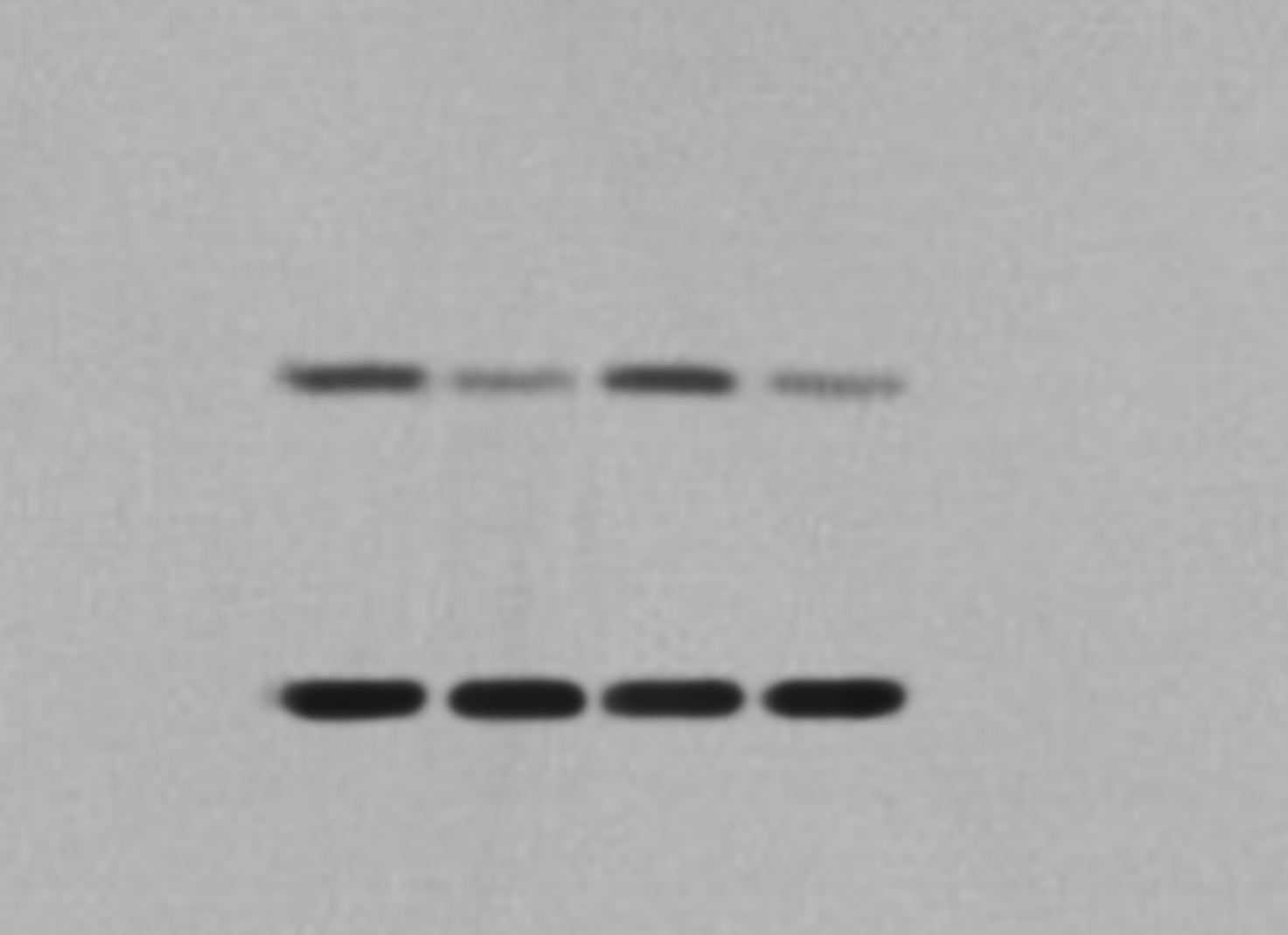

Supplement: S1 File — (ZIP) [file pone.0162754.s001.zip › western blotting/Fig4E-2.tif]

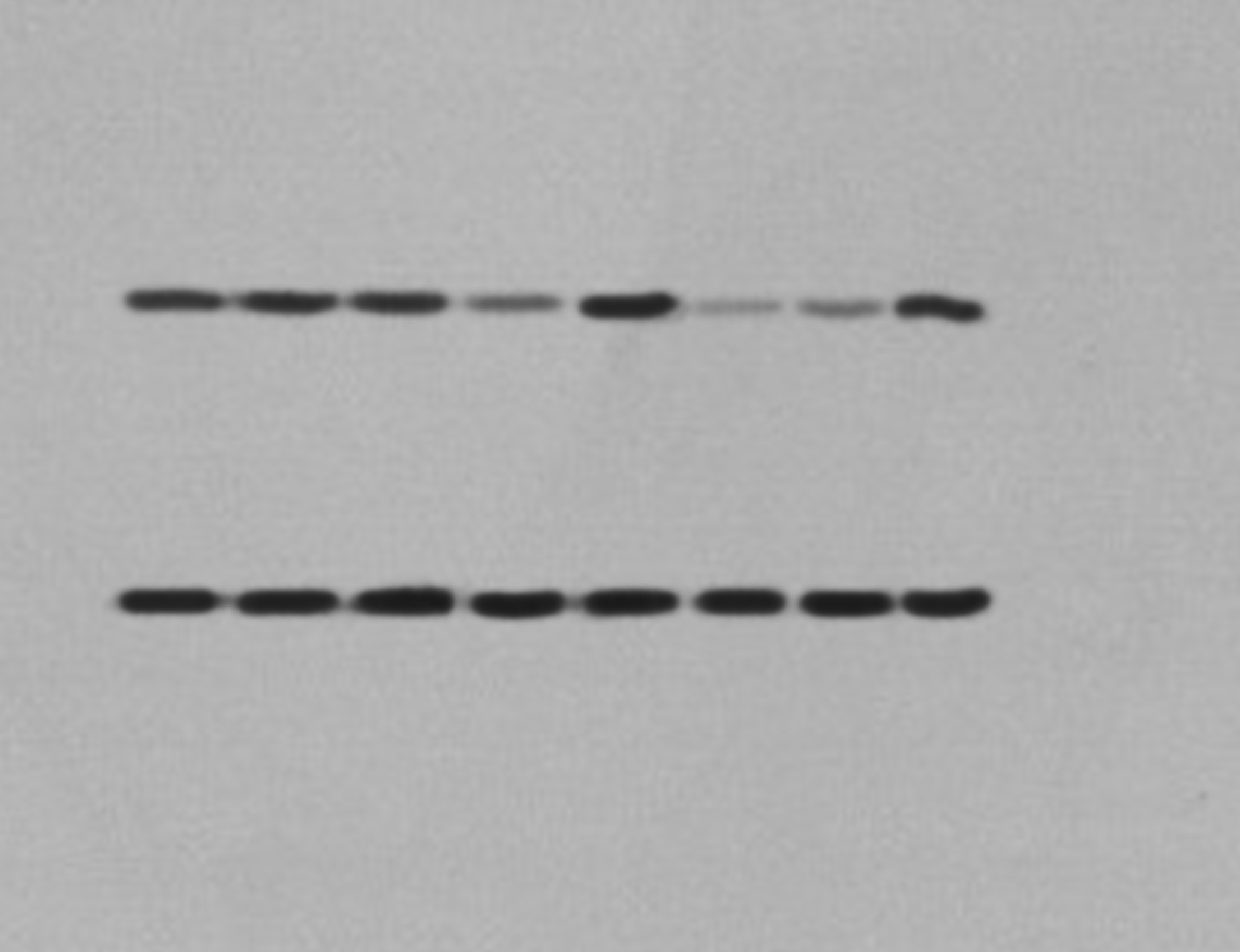

Supplement: S1 File — (ZIP) [file pone.0162754.s001.zip › western blotting/Fig5A.tif]

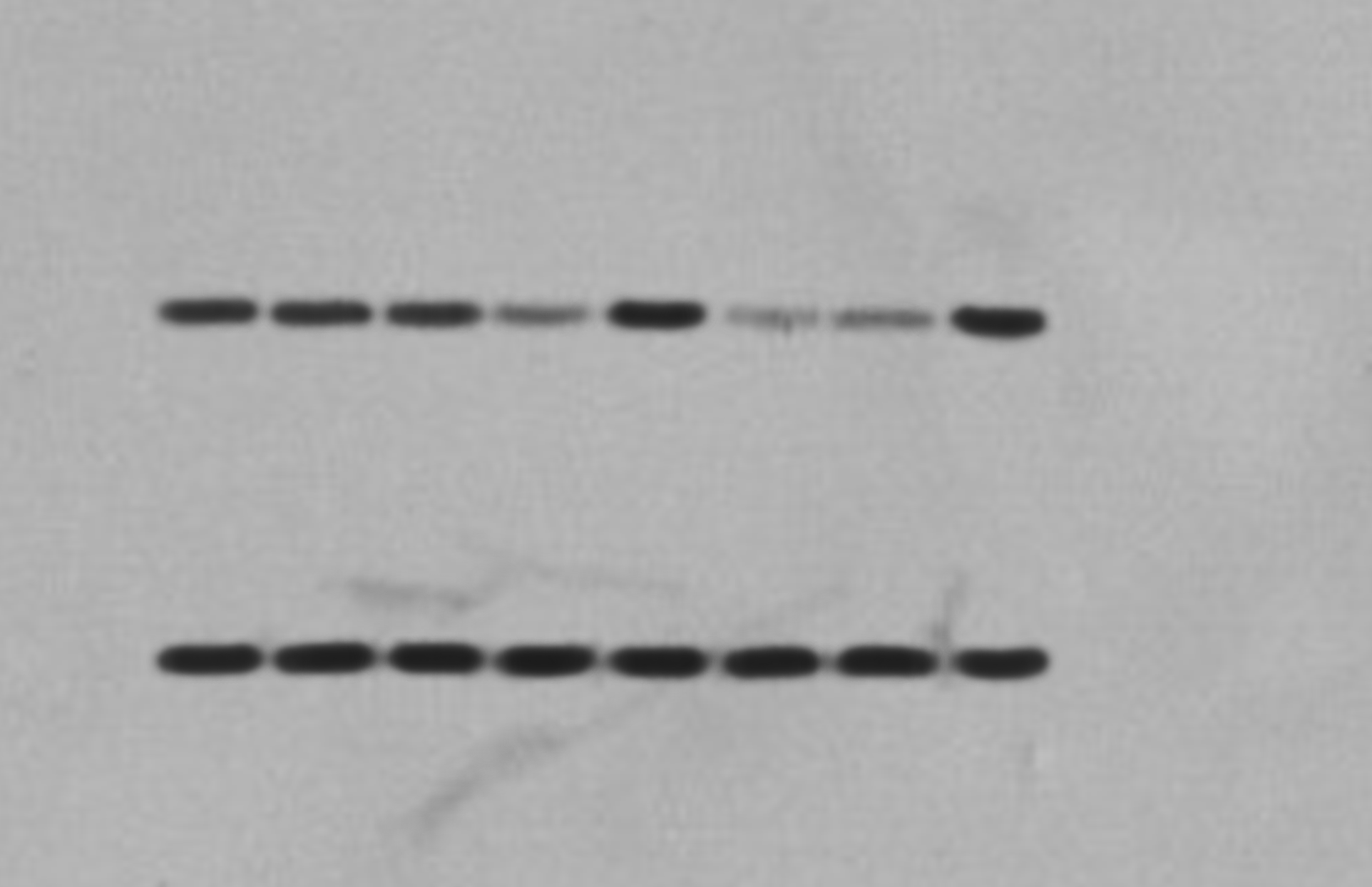

Supplement: S1 File — (ZIP) [file pone.0162754.s001.zip › western blotting/Fig5B.tif]

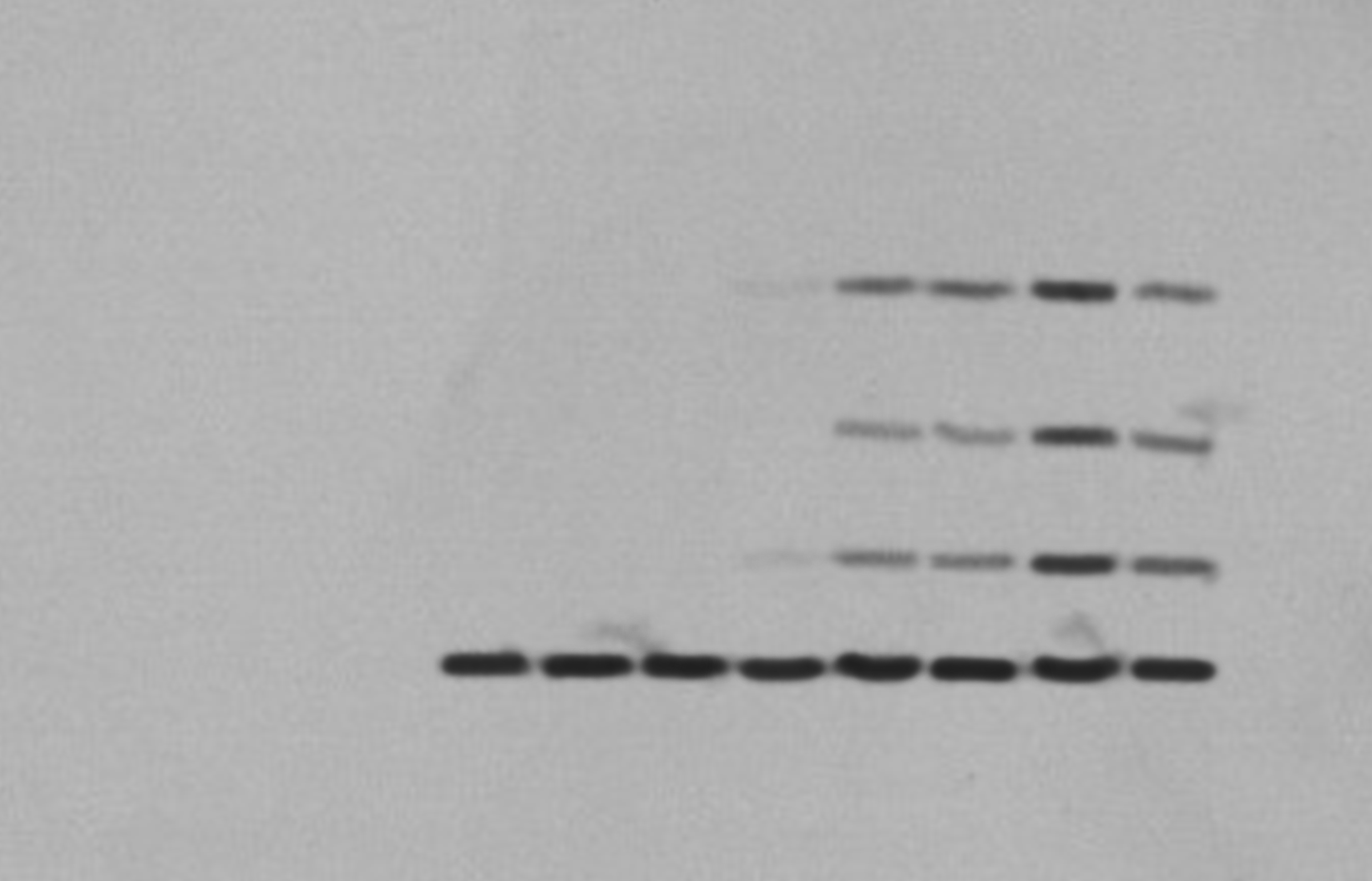

Supplement: S1 File — (ZIP) [file pone.0162754.s001.zip › western blotting/Fig6B-1.tif]

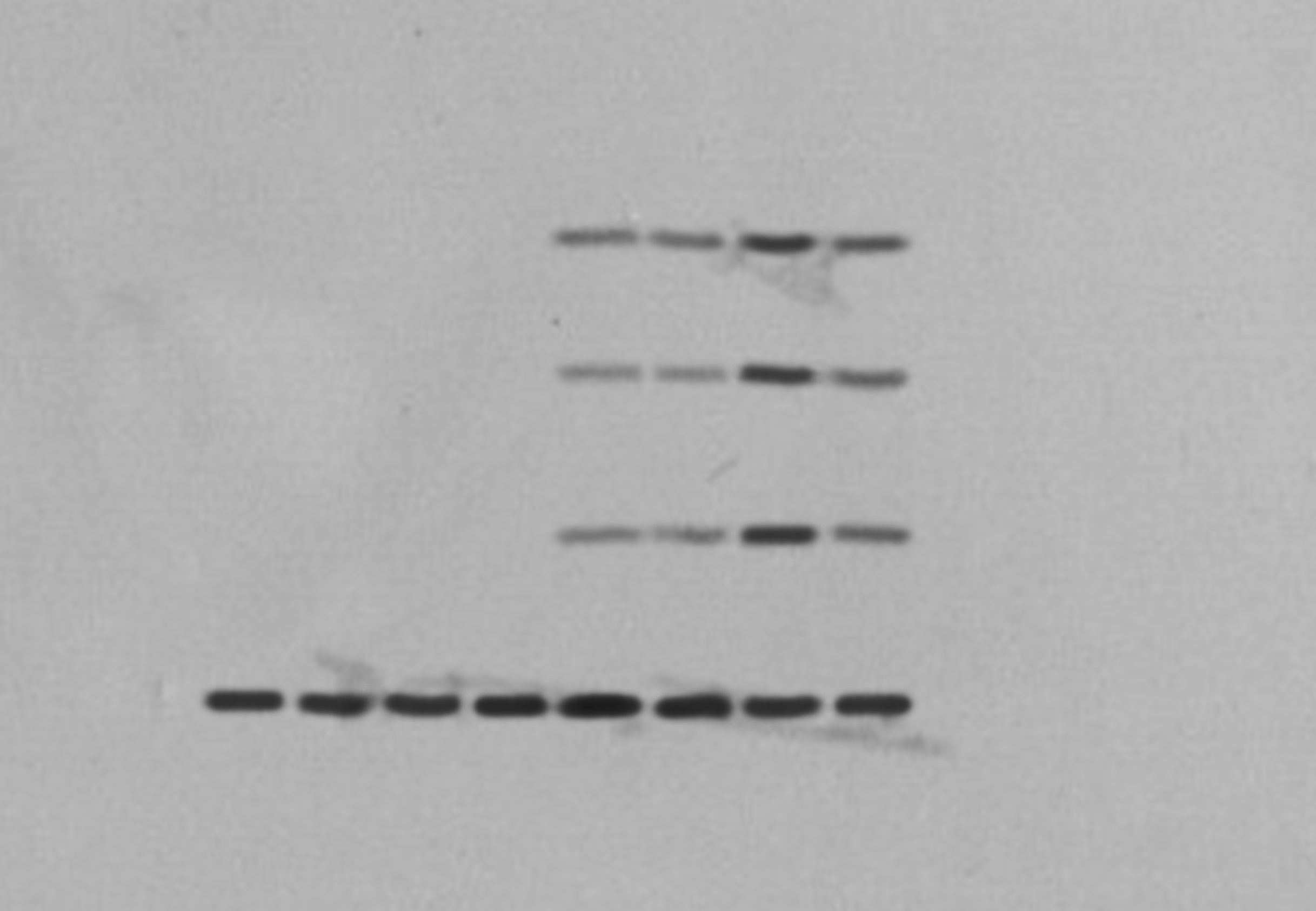

Supplement: S1 File — (ZIP) [file pone.0162754.s001.zip › western blotting/Fig6B-2.tif]
